# Pliocene cooling enhanced by flow of low-salinity Bering Sea water to the Arctic Ocean

**DOI:** 10.1038/ncomms8587

**Published:** 2015-06-29

**Authors:** Keiji Horikawa, Ellen E. Martin, Chandranath Basak, Jonaotaro Onodera, Osamu Seki, Tatsuhiko Sakamoto, Minoru Ikehara, Saburo Sakai, Kimitaka Kawamura

**Affiliations:** 1 Department of Geological Sciences, University of Florida, Gainesville, Florida 32611, USA; 2 Graduate School of Environmental Studies, Nagoya University, Furo-cho, Chikusa-ku, Nagoya 464-8601, Japan; 3 Japan Agency for Marine-Earth Science and Technology, 2-15 Natsushima-cho, Yokosuka 237-0061, Japan; 4 Institute of Low Temperature Science, Hokkaido University, N19W8, Kita-ku, Sapporo, Hokkaido 060-0819, Japan; 5 Center for Advanced Marine Core Research, Kochi University, B200 Monobe, Nankoku 783-8502, Japan

## Abstract

Warming of high northern latitudes in the Pliocene (5.33–2.58 Myr ago) has been linked to the closure of the Central American Seaway and intensification of North Atlantic Deep Water. Subsequent cooling in the late Pliocene may be related to the effects of freshwater input from the Arctic Ocean via the Bering Strait, disrupting North Atlantic Deep Water formation and enhancing sea ice formation. However, the timing of Arctic freshening has not been defined. Here we present neodymium and lead isotope records of detrital sediment from the Bering Sea for the past 4.3 million years. Isotopic data suggest the presence of Alaskan glaciers as far back as 4.2 Myr ago, while diatom and C_37:4_ alkenone records show a long-term trend towards colder and fresher water in the Bering Sea beginning with the M2 glaciation (3.3 Myr ago). We argue that the introduction of low-salinity Bering Sea water to the Arctic Ocean by 3.3 Myr ago preconditioned the climate system for global cooling.

## Abstract (web summary)

Late Pliocene cooling led to the glaciation of the Northern Hemisphere, yet its trigger remains unclear. Here, the authors present neodymium and lead isotope records from the Bering Sea, and propose that the introduction of low-salinity water into the Arctic Ocean preconditioned Pliocene cooling.

## Graphical abstract

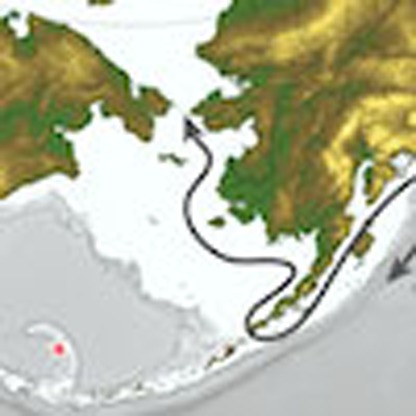

The period from 4.4–4.0 million years ago (Myr ago) was the warmest interval within the Pliocene epoch and was characterized by global mean temperatures ∼3 °C warmer than today^1^,^2^. The subsequent cooling throughout the late Pliocene culminated in the late-Pliocene climate transition (LPCT, 3.6–2.4 Myr ago), which led to major Northern Hemisphere glaciation at 2.73 Myr ago^3^,^4^. There is evidence for restricted, local glaciers on Arctic continents during the Pliocene warmth^5^; however, the evolution of these glaciers during the LPCT is poorly constrained, as is the role of the Central American Seaway (CAS)^6^. Tectonic shoaling of the CAS may have reached a critical threshold between ∼4.7 and 4.2 Myr ago^6^,^7^,^8^,^9^ that restricted Pacific–Caribbean surface-water exchange and redirected warm, saline waters from the equatorial Atlantic to the North Atlantic. These changes in the ocean circulation system are believed to contribute to an initial intensification of the Atlantic Meridional Overturning Circulation (AMOC) and warming of the North Atlantic prior to 3.6 Myr ago^9^,^10^,^11^,^12^. Yet, there is no consensus regarding what processes reduced the intensification of the AMOC and led to the late-Pliocene global cooling.

Modelling results suggest increased moisture supply (that is, snowfall) related to the increased AMOC was insufficient to produce the major glaciation of Greenland^12^. Alternative explanations emphasize increased freshwater inputs to the Arctic Ocean (AO) that could have generated lower sea-surface salinities (SSSs), leading to sea ice formation, increased ice-albedo effects and reduced heat exchange between the ocean and atmosphere^6^,^7^. The proposed source for this freshwater is the enhanced flow of Siberian rivers, driven by increased moisture transport to Eurasia by the AMOC^7^. Another possible source is low-salinity Bering Sea water^6^,^13^,^14^,^15^. The influence of Bering Sea water on the AO during the LPCT is particularly relevant because the flow direction through the Bering Strait is believed to have shifted from southward to its present northward direction at 3.6 Myr ago in response to changes in the steric height gradient between the North Pacific and AO, generated by closure of the CAS^11^,^13^. The freshwater influx to the AO today is derived from river discharge (38%), inflow of low-salinity Bering Sea water (30%) and net precipitation (24%)^16^,^17^. Thus, the amount of freshwater transported from the Bering Sea into the AO currently influences the strength of deep-ocean convection in the North Atlantic^18^,^19^,^20^. An excess influx of freshwater from the Bering Sea to the AO during the Pliocene may have compounded the effect of Siberian River inputs on the stability of the AMOC, resulting in cooling during the LPCT^6^.

Low salinities in the Bering Sea today are primarily derived from the glacier-fed Alaska Coastal Current (ACC)^21^ (Fig. 1). Therefore, Alaskan mountain glacier build-up and seasonal melting of ice should be necessary for establishing low salinities in the Bering Sea; however, the Pliocene evolution of this glacial input has been difficult to track due to limited records of the glacial history of Alaska and past surface-water properties in the Bering Sea. Here we generate Pliocene records of sediment provenance and surface-water properties in the Bering Sea to demonstrate the timing of cryospheric evolution in the Bering/Alaskan region and clarify the relationship between flow of low-salinity Bering Sea water to the AO and the late-Pliocene global cooling.

## Results

### Surface-water properties and Nd and Pb isotope records

In this study, we interpret the relative abundance of tetraunsaturated C_37_ alkenones to total C_37_ alkenones (% C_37:4_ alkenone) as a proxy for temperature and/or salinity, with higher percentages reflecting cool and/or freshwater masses^22^,^23^. The percentage of ‘sea-ice related' diatoms is the relative abundance of *Thalassiosira antarctica* resting spores, *Fragilariopsis cylindrus* and *Fr. oceanica* to the total number of diatoms^24^. Today, these diatoms are common in surface sediment from the Bering Sea shelf and slope, where sea ice expands seasonally, but they are absent or found in low abundances in surface sediments in the central Bering Sea, where sea ice is less common^25^. An additional proxy is the percent of *Actinocyclus* spp., which is a species that is abundant in surface water with meltwater-related stratification^24^,^26^.

**Figure 1:**
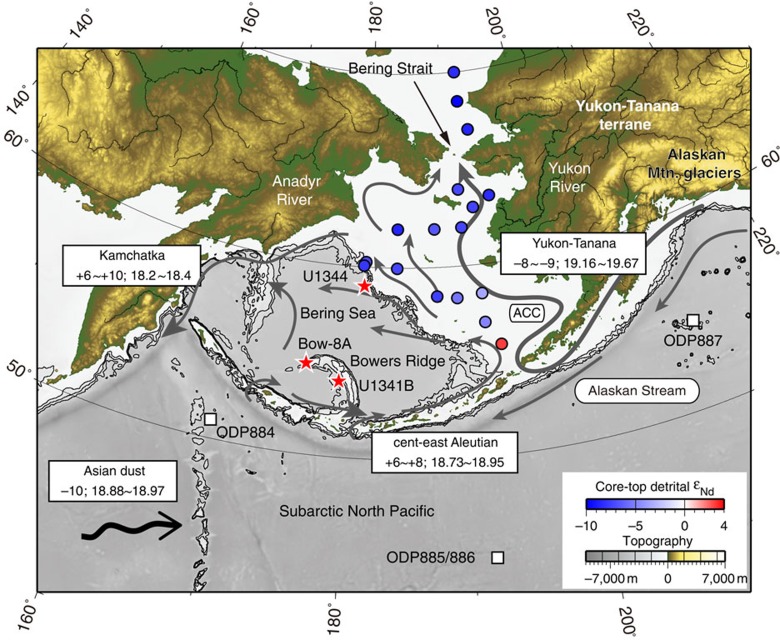
Locations of studied sediment cores and major surface currents. The Bering Strait is narrow (∼85 km wide) and shallow (∼50 m deep) at the far northern end of the Pacific Ocean that connects to the Arctic Ocean (AO). Glacial meltwater provides a substantial portion of the total freshwater runoff into the Alaska Coastal Current (ACC), and the low-salinity ACC flows to the AO. Grey arrows indicate subsurface flow at 40 m (ref. 37). On the map, numbers in the white boxes represent the *ɛ*_Nd_ value and the ^206^Pb/^204^Pb ratio of detrital materials in the source regions. Isotopic data are compiled from the GEOROC database^38^ and from the literature (see references in the figure caption of Fig. 3). Coloured circles represent sampling sites and their detrital *ɛ*_Nd_ values of surface sediments^34^. Red stars (this study) and white squares^30^,^40^,^54^ are the locations of core sites cited in this study. The black arrow represents the transport of Asian dust. Bathymetry and land topography are also shown.

**Figure 2:**
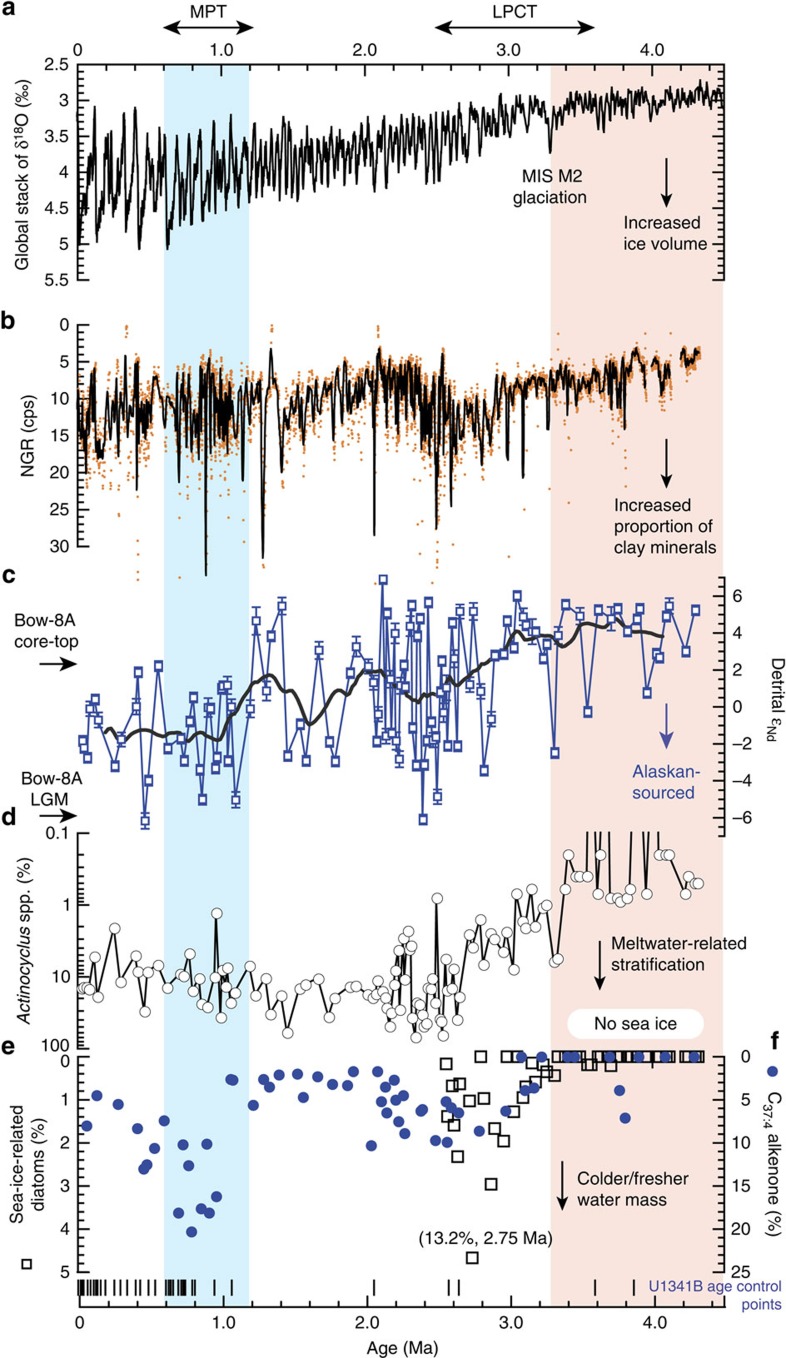
Multi-proxy records from Bering Sea Site U1341B for the past 4.3 Myr ago. (**a**) Global stack of δ^18^O record^4^. Records of (**b**) natural gamma radiation (NGR), (**c**) detrital Nd isotope values, (**d**) % *Actinocyclus* spp^24^., (**e**) % sea-ice-related diatoms^24^ and (**f**) % C_37:4_ alkenones from U1341B in the Bering Sea. The thick line in **c** represents the 400-kyr running mean, and the error bars correspond to 2*σ*_μm_ error (see details in Supplementary Table 1). From 4.3 to 3.3 Myr ago, there is no evidence of sea-ice-related diatoms in the southern Bering Sea. Some minor increases in % sea-ice-related diatoms before 3.3 Myr ago were not significant relative to error. These multi-proxy records from the Bering Sea highlight that the marine isotope stage (MIS) M2 glaciation at 3.3 Myr ago marks the beginning of the long-term trend towards cooling/freshening, meltwater-related stratification, expansion of sea ice and increased proportion of clay minerals in the southern Bering Sea. LPCT and MPT are Late-Pliocene climate transition and Mid-Pleistocene transition, respectively.

At IODP (Integrated Ocean Drilling Program) Site U1341B (Expedition 323) located at Bowers Ridge in the southern Bering Sea (Fig. 1; Methods; Supplementary Fig. 1; refs 27, 28), all three of these surface-water proxies record gradual increases beginning around the time of MIS (marine isotope stage) M2 glaciation at 3.3 Myr ago and continuing to 2.75–2.5 Myr ago (Fig. 2d–f),[Fig f3] indicating cooling and/or freshening and increased meltwater-related stratification. The highest % C_37:4_ alkenone values are observed during the mid-Pleistocene transition (MPT), probably reflecting additional cooling and/or freshening (Fig. 2f). The low-resolution % C_37:4_ alkenone record in the Bering Sea has remarkable similarities to a high-resolution % C_37:4_ alkenone record from Site 882 in the subarctic western North Pacific^29^, which also records the highest values during the MPT and stable, lower values prior to 3.4 Myr ago (Figs 2f and 4e). The lack of correlation between % C_37:4_ alkenones and % *Actinocyclus* spp. during the MPT is attributed to higher percentages in *T. trifulta* group and *T. antarctica* resting spores^24^, which prefer cold water and reduce the % *Actinocyclus* spp.

The detrital *ɛ*_Nd_ values from Site U1341B span a wide range from –6.2 to +6.9 *ɛ*_Nd_ over the past 4.3 Myr ago, with large, rapid fluctuations from 2.8 to 2.0 Myr ago (Fig. 2c). The *ɛ*_Nd_ variability was confirmed by replicate analysis of some of the samples with high and low *ɛ*_Nd_ values (Supplementary Table 1). Further, similar highly variable detrital *ɛ*_Nd_ values are documented in glacial–interglacial sediments at Bow-8A (–6.1 to +2.2 *ɛ*_Nd_; Supplementary Fig. 2) and at Site 884 (–1.2 to +8.6 *ɛ*_Nd_; ref. 30) (Fig. 1). These highly variable detrital *ɛ*_Nd_ values suggest regional glacial–interglacial shifts of sediment sources and/or sediment transport processes. A lower-resolution detrital lead (Pb) isotope record (*n*=29) also shows similar high variability (Supplementary Fig. 3; Supplementary Table 1). The long-term *ɛ*_Nd_ record (400-kyr running mean) demonstrates a marked shift from more radiogenic to less radiogenic values across the LPCT starting at 3.0 Myr ago and during the MPT starting at 1.2 Myr ago, coherent with the % C_37:4_ alkenone record (Fig. 2c,f). Further, the pronounced glacial MIS M2 is marked by the lower detrital *ɛ*_Nd_ value, together with the increases in % sea-ice-related diatoms and % *Actinocyclus* spp.

### Sediment provenance

Detrital material is delivered to the Bering Sea from continental sources via rivers, ice and wind, and distributed by surface and deep currents. Today, the Yukon River, the largest river in the Bering Sea, provides >63% (55 Mt yr^−1^)^31^ of the total sediment load to the Bering Sea in association with abundant freshwater discharge (6,400 m^3^ s^−1^)^32^ as it drains portions of the glaciated Alaska Range, Wrangell Mts and St Elias Mts in Alaska (Fig. 1; Supplementary Note 1). Approximately half of the annual suspended sediment exported via the Yukon River occurs during spring season snowmelt and the other half occurs during summer–autumn season glacier melt^33^. The meltwater river plumes, and counterclockwise surface currents in the Bering Sea distribute these Yukon River detrital sediments over long distances and a wide area of the eastern Bering Sea shelf^34^,^35^ (Fig. 1).

The Alaskan material stored on the Bering Sea shelf is transported to Bowers Ridge by the southward-flowing current. Thus, despite the distance from the Yukon outflow to Bowers Ridge, potential detrital sources at Site U1341B in the southern Bering Sea include the Yukon-Tanana terrane, Aleutian arc and aeolian dust from the Asian continent. Aeolian sources from Alaska and the Anadyr River detrital material might be less significant because the core site is not downwind from Alaska^36^, and the detrital flux from the Anadyr River is a small (2 Mt yr^−1^)^30^ and is restricted to the northern shelf margins by the southwestward-flowing Kamchatka current^37^, respectively. The three primary detrital sources, Yukon-Tanana terrane, Aleutian arc and Asian dust, have very distinct Nd and Pb isotopic signatures; Yukon-Tanana terrane Alaskan material has *ɛ*_Nd_ values of –8 to –9 and ^206^Pb/^204^Pb values of 19.16–19.67 (refs 30, 38, 39), Aleutian arc material has *ɛ*_Nd_ values of +6 to +8 and ^206^Pb/^204^Pb values of 18.73–18.95 (refs 30, 38) and Asian dust has *ɛ*_Nd_ values ca. –10 and ^206^Pb/^204^Pb values of 18.88–18.97 (ref. 40, 41, 42) (Fig. 1). Therefore, these isotope data will be sensitive monitors of provenance for the southern Bering Sea sediments.

**Figure 3 f3:**
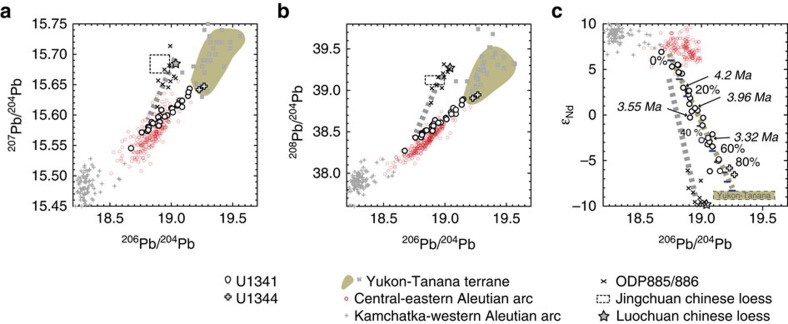
Pb and Nd isotope cross-plots for U1341B detrital sediments and potential local source rocks. (**a**) ^206^Pb/^204^Pb versus ^207^Pb/^204^Pb, (**b**) ^206^Pb/^204^Pb versus ^208^Pb/^204^Pb and (**c**) ^206^Pb/^204^Pb versus *ɛ*_Nd_ isotope plots. Core U1344 (open cross) was retrieved from the northern Bering slope near the Yukon River, and thus detrital sediments deposited during the LGM (last glacial maximum) and the last deglaciation are representative of Yukon River-delivered Alaskan detrital material. U1341B detrital sediments of Nd and Pb isotopes (open circle) represent a binary mixing line (coloured dashed line in **c**) showing a mixture of sediment from the Aleutian and Alaskan sectors^30^,^38^,^39^, whereas detrital sediments at Site 885/886 (× -mark, ref. 40) represent a linear array (grey dashed line in **a–c**) connecting Chinese loess and central–eastern Aleutian Arc sectors^38^,^41^,^42^. The coordinates of points (bar) on the Aleutian-Yukon (Alaska) mixing line (**c**) represent the proportion of the Alaskan detrital material (Methods). Detrital *ɛ*_Nd_ record from U1341B represents four pulses that produced progressively less radiogenic *ɛ*_Nd_ values prior to 3.3 Myr ago, and these data are marked with the ages in **c**. Although detrital materials from the Yukon-Tanana terrane have a wide range of ^206^Pb/^204^Pb ratios, the average ^206^Pb/^204^Pb ratio of Alaskan-sourced material is assumed to be 19.25 for the mixing line (Methods and Supplementary Table 3).

Nd and Pb isotopic compositions of detrital sediments from U1341B create linear arrays for isotope plots of ^206^Pb/^204^Pb versus ^207^Pb/^204^Pb, ^206^Pb/^204^Pb versus ^208^Pb/^204^Pb and ^206^Pb/^204^Pb versus *ɛ*_Nd_ which connect Aleutian arc material and Yukon-Tanana terrane Alaskan material (Fig. 3a–c). In contrast, data from Site 885/886 in the subarctic North Pacific^40^ create a distinct linear array that connects central–eastern Aleutian Arc materials and Chinese loess (Figs 1 and 3a–c). These data show that Site 885/886 is marked by higher fluxes of the Asian dust and lower detrital fluxes from the Aleutian arcs than U1341B. However, the Asian dust fluxes estimated at Site 885/886 for the past 5 Myr ago (8–122 μg cm^−2^ yr^−1^; ref. 40) and the fluxes observed in the modern North Pacific (22–100 μg cm^−2^ yr^−1^; ref. 43) are negligible compared with the higher mass accumulation rates of detrital materials for U1341B (8 mg cm^−2^ yr^−1^, assuming 14 cm kyr^−1^ as the average sedimentation rate, 40 wt% of detrital material content and 1.4 g cm^−3^ of bulk sediment density^28^). Thus, we interpret the detrital sediments of U1341B as a mixture dominated by sediments from the Aleutian and Alaskan sectors throughout the past 4.3 Myr ago.

Prior to MIS M2 at 3.3 Myr ago, most of the detrital Nd isotope data plot between +5 and +6 *ɛ*_Nd_ (Fig. 2c), suggesting that sedimentation at the site was dominated by local Aleutian sources. However, there are four pulses that produced progressively less radiogenic values. These occurred at 4.2 Myr ago, 3.96 Myr ago, 3.55 Myr ago, and the MIS M2 glaciation (3.3 Myr ago). A simple binary mixing model estimates that Alaskan-sourced detrital materials accounted for 13% of the detrital sediment at 4.2Myr ago, 26% at 3.96Myr ago, 32% at 3.55Myr ago and 48% at 3.32 Myr ago (Methods), indicating that the proportion of Alaskan inputs increased in progressively younger samples.

### Transport of Alaskan detrital material to Bowers Ridge

Core-top late-Holocene sediment from core Bow-8A at Bowers Ridge located near the Aleutian arc has an *ɛ*_Nd_ value of +2.2 (Fig. 1; Supplementary Fig. 2). Simple binary mixing (Methods) suggests the core-top sediment is predominantly composed of central–eastern Aleutian material with a minor component of Alaskan material (18%). This Yukon River-derived Alaskan material stored on the Bering Sea shelf may have been transported to Bowers Ridge by the southward-flowing current. In contrast, during the last deglaciation and the last glacial maximum (LGM), detrital sediments at Bow-8A have less radiogenic *ɛ*_Nd_ values of –6.1 to –4.1 (Supplementary Fig. 2), which translates to a much greater contribution from Alaskan sources (60–78%). A measured decrease in diatom δ^18^O in the northeast Bering Sea during the deglacial is believed to reflect a large flux of meltwater from retreating alpine glaciers in Alaska^44^, which may have transported some of the detrital sediment. Evidence from diatom assemblages suggests the presence of drift ice at northern Bowers Ridge at the LGM^45^. Given these results, it appears that meltwater plumes and sea ice rafting were responsible for transporting a large amount of Alaskan detrital materials from the prograded mouth of the Yukon River towards Bowers Ridge during deglacial and glacial periods, respectively.

During glacial periods when sea level fell, the closed Bering Strait (currently 50 m deep) would also have altered both surface and intermediate water circulation in the Bering Sea^45^,^46^. Yet, the tight coupling between detrital Nd isotopes and foraminiferal δ^18^O records from Bow-8A within the Bering Sea (Supplementary Fig. 2) and Site 884 in the North Pacific south of the Aleutian Arc^30^ suggests delivery of Alaskan detrital material was driven by a process beyond the Bering Sea rather than internal variations in circulation within the Bering Sea caused by the closure of the Bering Strait. Instead, changes driven by ice volume, such as the location of Alaskan river outflow, the extent of sea ice formation and the volume of meltwater, appear to provide better explanations of observed changes in the composition of detrital material during glacial–interglacial cycles.

**Figure 4:**
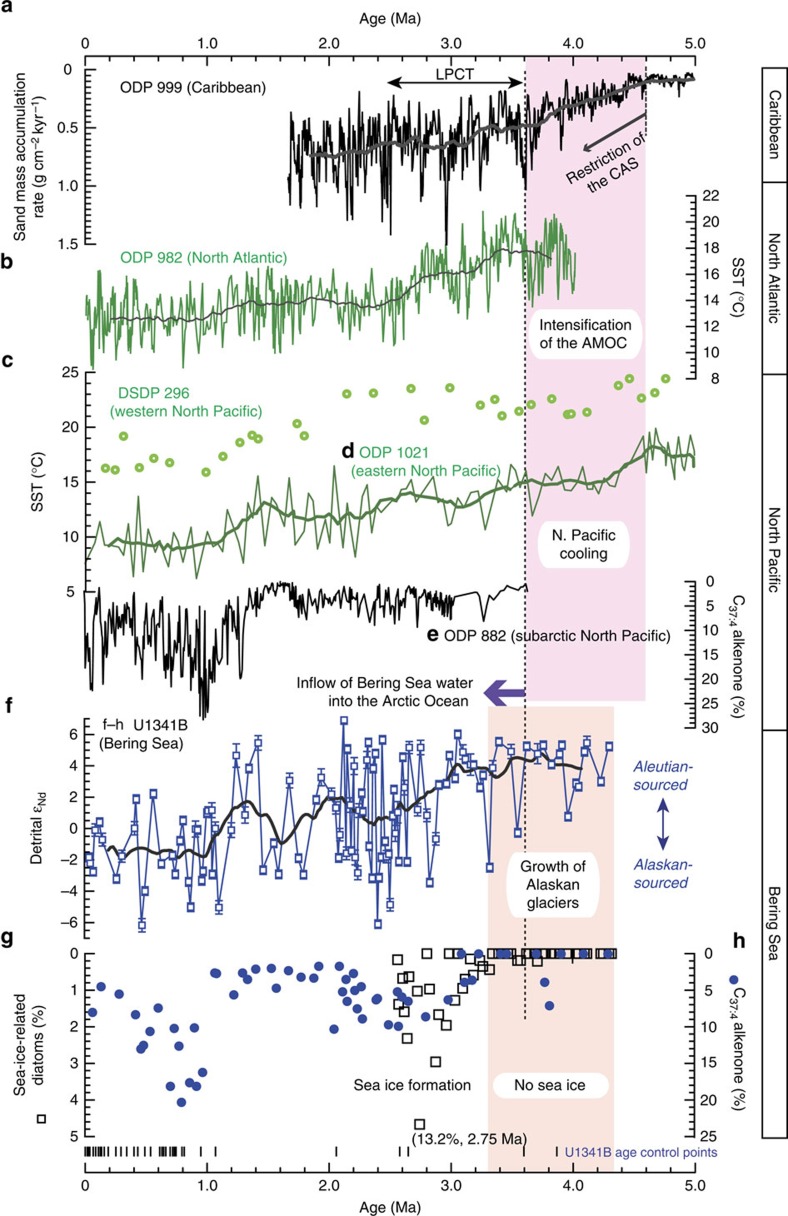
Comparison of global and regional data from the Pliocene–Pleistocene. (**a**) Sand mass accumulation rate in the Caribbean Sea^9^, (**b**) Alkenone-derived SST records in the North Atlantic^60^, (**c**) Winter SST records based on transfer function methods of planktic foraminifera assemblages in the western North Pacific^51^, (**d**) Alkenone-derived SST records in the eastern North Pacific^52^, (**e**) C_37:4_ alkenone abundance from the subarctic western North Pacific^29^, (**f–h**) Records of detrital Nd isotopes, sea-ice-related diatom abundance^24^ and C_37:4_ alkenone abundance from Bering Sea Site U1341B. Restriction of the CAS (Central American Seaway) started at 4.6–4.7 Myr ago^8^,^9^. The presence of Alaskan mountain glaciers was as far back as 4.2 Myr ago, and the flow direction of Bering Strait throughflow has shifted from southward to its present northward direction at 3.6 Myr ago^13^ (dashed line). Introduction of low-salinity Bering Sea water into the AO might have started by at least 3.3 Myr ago. Thick lines in **a**,**b**,**d** and **f** represent the 400-kyr running mean.

In the early Pliocene, most of the Nd isotopic data are centred on +5 to +6 *ɛ*_Nd_, higher than the Bow-8A core-top value (+2.2 *ɛ*_Nd_) (Fig. 2c), and there is no evidence of sea-ice-related diatoms from 4.3 to 3.3 Myr ago^24^ (Fig. 2e). These findings indicate that the southern Bering Sea was relatively warm and there was insufficient ice or glacial melt to transport Alaskan detrital material, consistent with the general view of Pliocene warmth^1^,^2^,^47^ (Fig. 4b–d). The relatively stable and radiogenic *ɛ*_Nd_ values observed from 4.3 to 3.3 Myr ago transition to an interval of high-amplitude variations and overall decreasing *ɛ*_Nd_ values across the LPCT from 3.3 Myr ago to the MPT starting at 1.2 Myr ago. This long-term shift of detrital *ɛ*_Nd_ coincides with (1) greater variability and a general increase in global ice volume (Fig. 2a) and (2) increasing natural gamma radiation in the core, indicating an increased proportion of clay minerals particularly during glacial intervals^48^ (Fig. 2b) and (3) increasing % C_37:4_ alkenones, indicating cooling and/or freshening of water masses^22^,^23^ (Fig. 2f). These proxies further support the interpretation of increasing glacial ice volume in the Bering/Alaskan region during the LPCT to MPT. The correlation between increased ice volume, increased proportions of clay minerals and lower detrital Nd isotopes argues that sea ice rafting and snow/glacier melt discharge from the Yukon River supplied a larger fraction of the Alaskan detrital material to Site U1341B for these periods.

## Discussion

Previous research on Gulf of Alaska sediments shows the presence of tidewater glaciers in southeastern Alaska since the Late Miocene, with more definitive evidence for glaciers based on the presence of dropstones beginning at 4.2–4.3 Myr ago^49^. The Pliocene detrital *ɛ*_Nd_ record from U1341B shows pulses of progressively greater inputs of Alaskan detrital material beginning as early as 4.2 Myr ago (Figs 2c and 4f). The first pulse of Alaskan detrital inputs that coincides with a slight increase in % sea-ice-related diatoms and a significant increase in % *Actinocyclus* spp. occurs during the MIS M2 glaciation at 3.3 Myr ago (Fig. 2c–e). This correlation indicates the first Alaskan glaciers large enough to produce associated meltwater and sea ice appeared at 3.3 Myr ago.

In contrast, other early pulses of Alaskan detrital material inputs at 4.2, 3.96 and 3.55 Myr ago lack a response in % C_37:4_ alkenones and % *Actinocyclus* spp. Although Alaskan mountain glaciers appear to have developed in the early Pliocene, the lack of coherent responses suggests that Alaskan glaciations were probably limited in size and early-glacial meltwater plumes were not large enough to produce stratification and freshening in the southern Bering Sea. However, initial deposition of Alaskan-sourced detrital material on the Bering Sea shelf may have been sourced by smaller glacial meltwater pulses and transported to Bowers Ridge by the southward-flowing current, as seen in the Holocene. The details of current processes that transported Alaskan detrital sediments to the Bowers Ridge remain uncertain, but an important point is that pulses of Alaskan detrital inputs identified by detrital Nd isotopes in the early Pliocene support growth of Alaskan mountain glaciers as a necessary precondition for the deposition of Alaskan-sourced detrital material on the Bering Sea shelf. Further, we also propose that increased volumes of melt with progressively larger glaciers from 4.2 Myr ago would have increased the depositional area of Alaskan-sourced detrital material on the Bering Sea shelf, resulting in progressively greater inputs of Alaskan detrital material to the Bowers Ridge.

Early-Pliocene evolution of Alaskan mountain glaciers (∼4.2–3.3 Myr ago) capable of producing a sedimentary signature of Alaskan material at Bowers Ridge may be related to a series of ocean–climate feedbacks triggered by the restriction of the CAS starting 4.6–4.7 Myr ago (Fig. 4a). A coupled ocean–atmosphere general circulation model found that meridional heat transport in the North Pacific weakened in response to reduced transport of saline water to the equatorial Pacific as the CAS shoaled^50^. This change in heat transport facilitated cooler air temperatures around the North Pacific and more snow accumulation over Alaska and western Canada^50^. Pronounced decreases in sea-surface temperature (SST) in the western subtropical and eastern North Pacific from ∼4.4 to 4.6 Myr ago^51^,^52^ (Fig. 4c,d) and persistent cooling of the Chinese Loess Plateau beginning ∼4 Myr ago^53^ support this modelling result. We acknowledge the lack of the detrital Nd isotopic record prior to 4.3 Myr ago limits our evaluation of the relationship between restriction of the CAS and growth of Alaskan mountain glaciers. However, terrigenous mass accumulation rates from ODP 887 in the Gulf of Alaska also increased from 4.55 Myr ago, with relatively higher values after 4.24 Myr ago^54^, again supporting a relationship between the sediment flux from Alaska and restriction of the CAS. On the basis of these data around Alaska, we propose that the growth of Alaskan mountain glaciers in the early Pliocene was accelerated by the reduction of northward heat transport in the North Pacific due to the restriction of the CAS at 4.6–4.7 Myr ago.

The evolution of a relatively fresh ACC is poorly constrained, but is presumably linked to the onset of Alaskan glaciation, which had started by at least 4.2 Myr ago. Smaller Alaskan mountain glaciers in the early Pliocene might have started to feed glacial meltwater to the coastal Bering Sea, but the combination of the small volume and the southward flow direction of Bering Strait throughflow limited the impact in the AO. Reversal of the Bering Strait throughflow at 3.6 Myr ago^11^,^13^ might be related to a change in the steric sea-level gradient between the Arctic and Bering Sea basins driven by increases in SSS in the North Atlantic and decreases in SSS in the North Pacific in response to restriction of the CAS. The resulting flow might have first introduced northern Bering Sea coastal water into the AO, but freshwater inputs from the Bering Sea to the AO were not significant enough to perturb the freshwater balance in the AO.

Our evidence for long-term cooling/freshening, meltwater-related stratification and expansion of sea ice in the southern Bering Sea by 3.3 Myr ago supports the establishment of more extensive ice on Alaska by this time, which enhances the likelihood that a fresher ACC developed in the eastern Bering Sea and delivered low-salinity water to the AO. Progressively larger fluxes of meltwater from progressively larger Alaskan mountain glaciers that developed from 3.3 to 2.8–2.5 Myr ago, as documented in C_37:4_ alkenone and *Actinocyclus* spp. records, would have further reduced salinity in ACC and ultimately in the AO.

Introduction of low-salinity Bering Sea water into the AO, starting by at least 3.3 Myr ago, would have perturbed the freshwater budget in the AO. Development of a low-salinity surface layer in the AO would have isolated the warmer, more saline intermediate waters from the atmosphere, and promoted expansion of sea ice in the North Atlantic that began at 3.2 Myr ago^55^,^56^ as well as freshening of the East Greenland Current between 3.25 and 3.16 Myr ago^14^. This scenario sets up northern high-latitude cooling through ice-albedo feedbacks. Ultimately, outflow of this low-salinity Arctic water to the North Atlantic would have weakened the ‘super conveyor' of North Atlantic deep water (4.0–3.0 Myr ago)^10^,^57^, further contributing to major continental glaciation in the Northern Hemisphere. Indeed, a recent coupled ocean–atmosphere climate model conducted under boundary conditions at 112 kyr supports this sequence of events, with introduction of low-salinity water from the Bering Sea to the AO perturbing the freshwater balance in the AO and the subpolar North Atlantic, muting the AMOC and leading to cooling in the Northern Hemisphere^20^. Even if the inflow of the low-salinity Bering Sea water into the AO was not the ultimate driver of the Pliocene climate cooling, Bering Strait throughflow of freshwater transport into the AO might have worked as an effective amplifier to cool the northern high latitudes starting at 3.3 Myr ago.

In summary, our Nd isotopic data highlight the presence of Alaskan detrital material at the Bowers Ridge as far back as 4.2 Myr ago. Transport of this material by sea ice and meltwater plumes supports development of Alaskan mountain glaciers in the early Pliocene; timing that coincides with proposed North Pacific cooling in response to restriction of the CAS. Increasing fluxes of meltwater from progressively larger Alaskan mountain glaciers from 3.3 to 2.8–2.5 Myr ago are indicated by the Nd isotopic data, as well as % *Actinocyclus* spp. and % C_37:4_ alkenones at Site U1341B. Introduction of low-salinity Bering Sea water into the AO starting by at least 3.3 Myr ago would have strengthened halocline development and sea-ice formation in the AO, leading to subarctic cooling/freshening from 3.2 to 3.3 Myr ago through ice-albedo feedbacks.

We argue that the multiple gateway events (restriction of the CAS and reversal of the Bering Strait throughflow) in the Pliocene established the glacier-fed, low-salinity Bering Sea water and its inflow into the AO, respectively. The effect of Bering Strait throughflow on freshwater transport into the AO during the early to late Pliocene should be tested quantitatively by numerical modelling to better understanding the outflow of low-salinity Arctic water to the North Atlantic and its impact on the stability of the AMOC in the Pliocene.

## Methods

### Sediment core samples

We analysed detrital Nd and Pb isotopes from sediment cores obtained at Site U1341B (54°02.00′N, 179°00.52′E, 2,140 m water depth) at Bowers Ridge in the southern Bering Sea, and at Site U1344 (59°03.02′N, 179°12.20′E, 3,173 m water depth) at the northern Bering slope during the IODP Expedition 323 (Fig. 1; ref. 27). Supplementary detrital Nd isotope data were also obtained from core Bow-8A (54°47′N, 176°55′E, 884 m water depth) near site U1341B during the R/V Hakuho-maru cruise KH99-3 (Fig. 1).

Sediments obtained from site U1341B are composed of a variable mixture of detrital, biogenic and authigenic components; there is a major contribution of biogenic opal and detrital clay/silt, and calcium carbonate content is generally low throughout the core (<1.0 wt%)^27^,^28^. The initial age model for Site U1341B was originally based on several paleomagnetic and siliceous microfossil datums that have been determined from an on-board study^27^,^28^. However, in this paper the age model has been updated using detailed onshore biostratigraphy^24^, magnetostratigraphy and foraminifera δ^18^O stratigraphy (Supplementary Fig. 1; Supplementary Table 2). The age–depth plot at Site U1341B was produced by assuming linear sedimentation rates between the age control points, and by extrapolating the sedimentation rate beyond the oldest diatom-based datum (3.87 Myr ago); this resulted in an estimated bottom sediment age of ∼4.3 Myr ago (Supplementary Fig. 1; Supplementary Table 2). We sampled U1341B at constant depths (4.5 and 7.5 m) in each core (*n*=115). Variations in the sedimentation rate produced apparent variations in the sample density (time intervals range from 5 to 116 kyr) of 115 Nd isotope data points plotted against age (Fig. 2c; Supplementary Fig. 1). Furthermore, we also generated Pb isotopic compositions of detrital sediments (*n*=29) from U1341B (Supplementary Fig. 3). Other detailed sedimentological and geochemical data relating to core U1341 can be seen in associated literature^24^,^48^,^58^.

Core U1344 is characterized by higher proportions of siliciclastic components than core U1341B. The last deglaciation and the LGM sediments of core U1344 show the Nd and Pb isotopic compositions (*ɛ*_Nd_=–5.8 and –6.6; ^206^Pb/^204^Pb=19.23 and 19.27) that are close to those of source rocks in the Yukon-Tanana terrane in Alaska (*ɛ*_Nd_=–9 to –8; ^206^Pb/^204^Pb=19.16 to 19.67; Fig. 3a–c).

### Detrital Nd and Pb isotopes

We leached bulk sediments (∼1.0 g) for 24 h on an orbital shaker at room temperature in 0.02 M hydroxylamine hydrochloride–25% Optima acetic acid, buffered to pH 4 with Optima sodium hydroxide. This reductive cleaning step also completely removed carbonate (av. 0.9 wt%, ref. 28) from the bulk sediment. Following centrifugation and triple rinses in ultrapure water, the samples were dried in an oven at 60 °C. The oxide-free bulk sediments (∼40 mg) were dissolved in a 7-ml Teflon vial by digestion in a concentrated Optima HF-HNO_3_ mixture (1:3 v/v) for ∼48 h on a hotplate (∼120 °C). Bulk rare-earth elements were separated by a primary column using cation-exchange resin (Mitsubishi diaion CK08P, 75–150 μm) and 1.7 N HCl as an eluent. Nd was then isolated by passing the REE aliquot through Eichrom Ln-spec resin (50–100 μm) with 0.25 N HCl as an eluent. Nd aliquots were dried and then redissolved in 2% HNO_3_ for analysis on a Nu Plasma MC-ICP-MS with a DSN-100 nebulizer at the University of Florida^59^. The total procedural blank for Nd is negligible (14 pg) compared with the amount of Nd in the samples. Pb aliquots were redissolved in 1 N seastar HBr and passed through Dowex 1-X8 (100–200 mesh) resin. Pb was collected in 20% Optima HNO_3_. The total procedural blank for Pb is 10–30 pg Pb and is negligible. Thallium (Tl)-spiked Pb concentrates dissolved in 2% HNO_3_ were adjusted for appropriate dilution to obtain a 2–5-V beam on ^208^Pb. Samples were aspirated through a DSN-100 nebulizer and Pb isotopes were measured by a Nu Plasma MC-ICP-MS. A Tl normalization technique was used for Pb isotope analyses. Long-term NBS 981 values analysed over several years at the University of Florida are ^206^Pb/^204^Pb=16.937±0.004 (2*σ*), ^207^Pb/^204^Pb=15.489±0.003 (2*σ*) and ^208^Pb/^204^Pb=36.695±0.008 (2*σ*).

### C_37:4_ alkenone abundance

Total lipids were extracted from 1–3.5 g of dried sediments with dichloromethane/methanol (95:5) using an accelerated solvent extractor (ASE 200, Dionex) at 100 °C and 1,000 p.s.i. for 10 min. The total extracts were separated into three fractions using silica gel column chromatography: aliphatic and aromatic hydrocarbons, aliphatic ketones (alkenones) and alcohol plus fatty acid fractions. The C_37_ alkenones were analysed with an HP6890 gas chromatograph (GC) equipped with an on-column injector-fused silica capillary column (CPSIL-5 CB, 50 m length × 0.32 mm inner diameter, film thickness of 0.12 mm), and a flame ionization detector. The GC oven temperature was programmed to increase from 80 to 180 °C at 10 °C min^−1^, and then from 180 to 310 °C at 5 °C min^−1^. The relative abundance of the tetraunsaturated C_37_ alkenones to total C_37_ alkenones (% C_37:4_) was defined as % C_37:4_=[C_37:4_]/([C_37:2_]+[C_37:3_]+[C_37:4_]) × 100.

### Isotope mass balance calculation

A simple isotope mass balance calculation is based on a two-end-member mixing model. If A and B are components containing different concentrations of element X, and if *R*^*X*^ is an isotope ratio of X, then the following equation can be obtained:


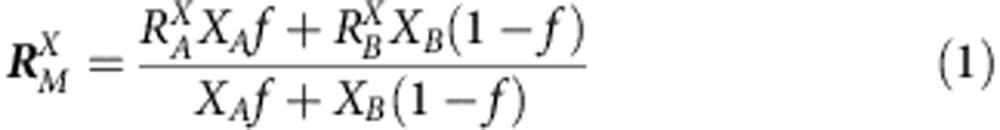


where 
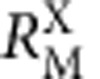
 is the isotope ratio of X in a mixture of components A and B, *X*_*A*_ and *X*_*B*_ are concentrations of X in A and B, respectively, and *f* is the weight fraction of A.

In our study, based on Nd and Pb isotope analyses, we consider Aleutian arc and Alaskan materials as end-members (see Fig. 3 and details in the text). The average values of Nd and Pb (^206^Pb/^204^Pb) isotopic compositions and their concentrations in the Aleutian and Alaskan materials were estimated from the GEOROC database^38^ and relevant literature^34^,^39^ (Supplementary Table 3). However, there were no available data for Pb concentrations in the Alaskan detrital material. Therefore, we evaluated a number of binary mixing curves that had different Pb concentrations (5, 10, 15 and 20 μg g^−1^) in Alaskan detrital material (Supplementary Fig. 4). The Nd–Pb isotopic compositions of the U1341B detrital sediments were then plotted around a mixing line with a Pb concentration of 10 μg g^−1^ (Supplementary Fig. 4). Therefore, we set a value of 10 μg g^−1^ as the Pb concentration in Alaskan-sourced detrital material, and then calculated the contribution from Alaskan-sourced detrital materials to the sediments at 4.2 (13%), 3.96 (26%), 3.55 (32%) and 3.32 Myr ago (48%).

## Additional information

**How to cite this article:** Horikawa, K. *et al.* Pliocene cooling enhanced by flow of low-salinity Bering Sea water to the Arctic Ocean. *Nat. Commun.* 6:7587 doi: 10.1038/ncomms8587 (2015).

## Supplementary Material

Supplementary Information Supplementary Figures 1-4, Supplementary Tables 1-3, Supplementary Note 1 and Supplementary References
