# Supplementary material for: Pliocene cooling enhanced by flow of low-salinity Bering Sea water to the Arctic Ocean
Source: Nat Commun. 2015 Jun 29;6:7587. doi: 10.1038/ncomms8587 (PMC4491831; doi:10.1038/ncomms8587)
Supplement: Supplementary Information — Supplementary Figures 1-4, Supplementary Tables 1-3, Supplementary Note 1 and Supplementary References [file ncomms8587-s1.pdf]

Supplementary Figures

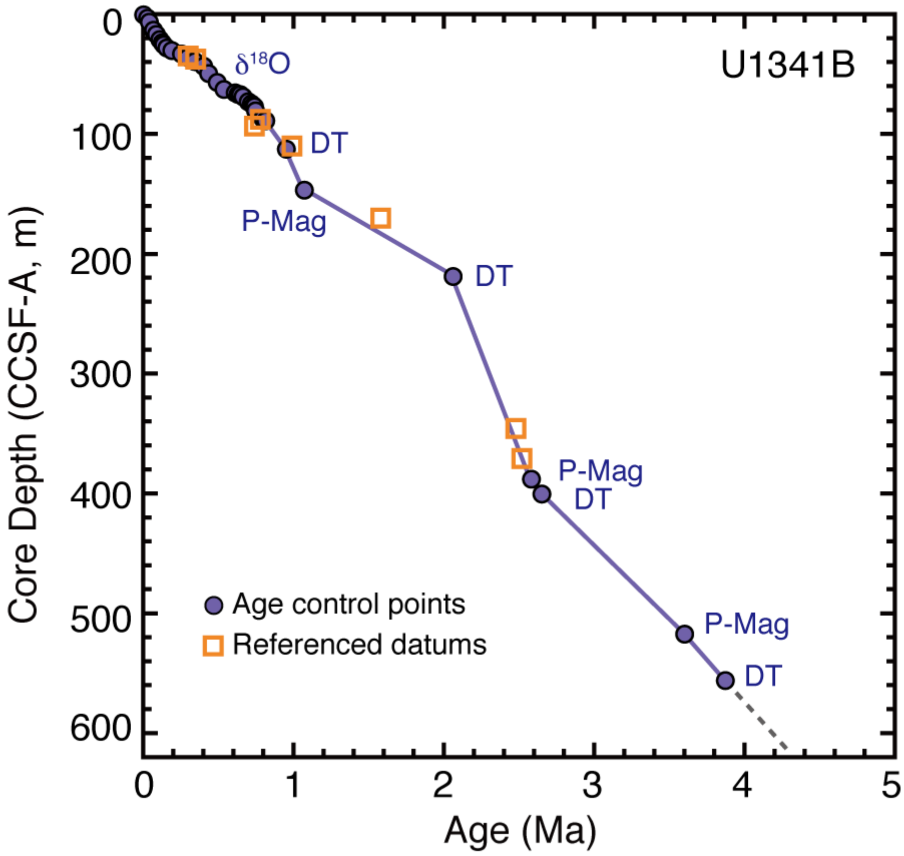

Supplementary Figure 1. Age-depth plot at Site U1341B. The age model is based on benthic foraminifera $\delta^{18}O$ and well-dated diatom and paleomagnetic datums (circles). This age-depth plot is consistent with ages estimated from other diatom, silicoflagellate, ebridian, and paleomagnetic datums (squares) that were not applied in our age model because of the relatively large age uncertainty (Supplementary Table 2). DT and P-Mag represent diatom and paleomagnetic datums, respectively.

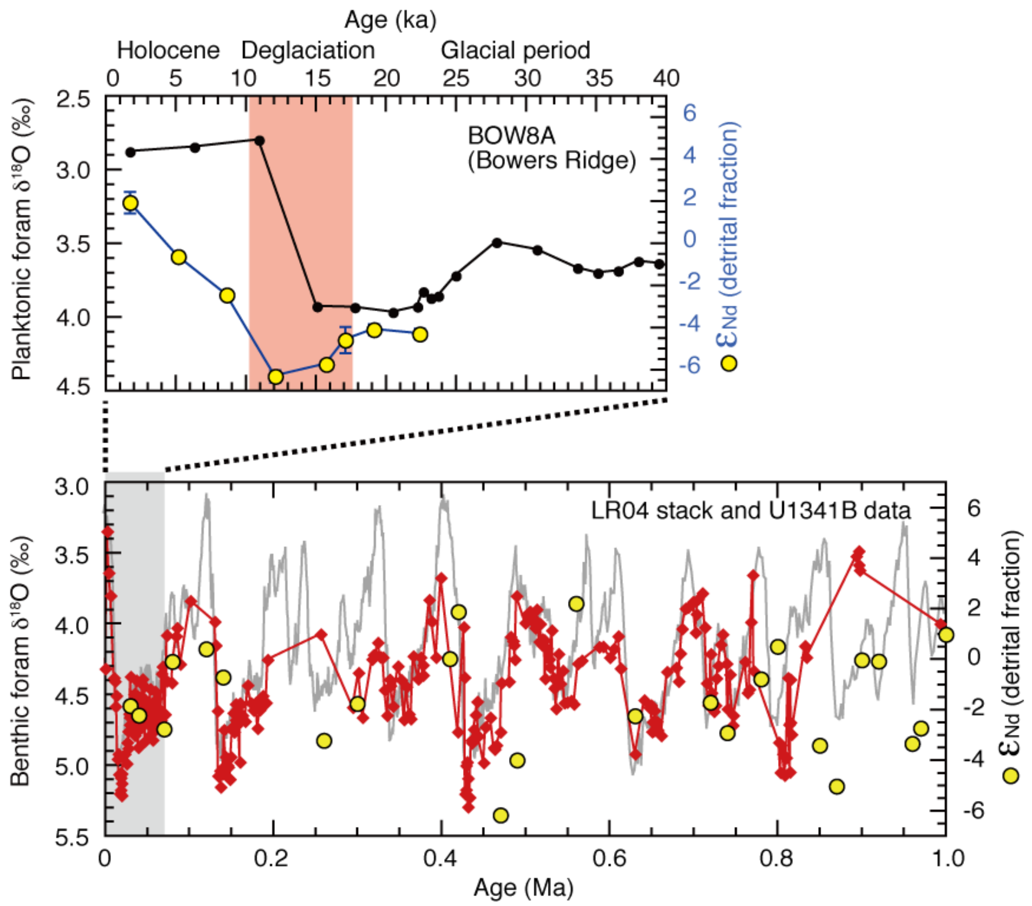

Supplementary Figure 2. Records of foraminifera $\delta^{18}O$ and detrital ε_{Nd} from Bowers Ridge sites (Bow-8A and U1341B) in the southern Bering Sea. Detrital ε_{Nd} values are more radiogenic during the Holocene and less radiogenic during the last deglaciation and the glacial period (upper panel) (the $\delta^{18}O$ record is from ref. 1 and some of the detrital Nd isotope data are obtained from ref. 2). The significant changes in detrital ε_{Nd} values can be seen in Site U1341B for at least the past 1.0 Myr, probably corresponding to higher interglacial values and lower glacial (deglacial) values. The record of U1341B benthic foraminifera $\delta^{18}O$ (red diamond) is tuned by the global stack of $\delta^{18}O$ curve[3] (gray).

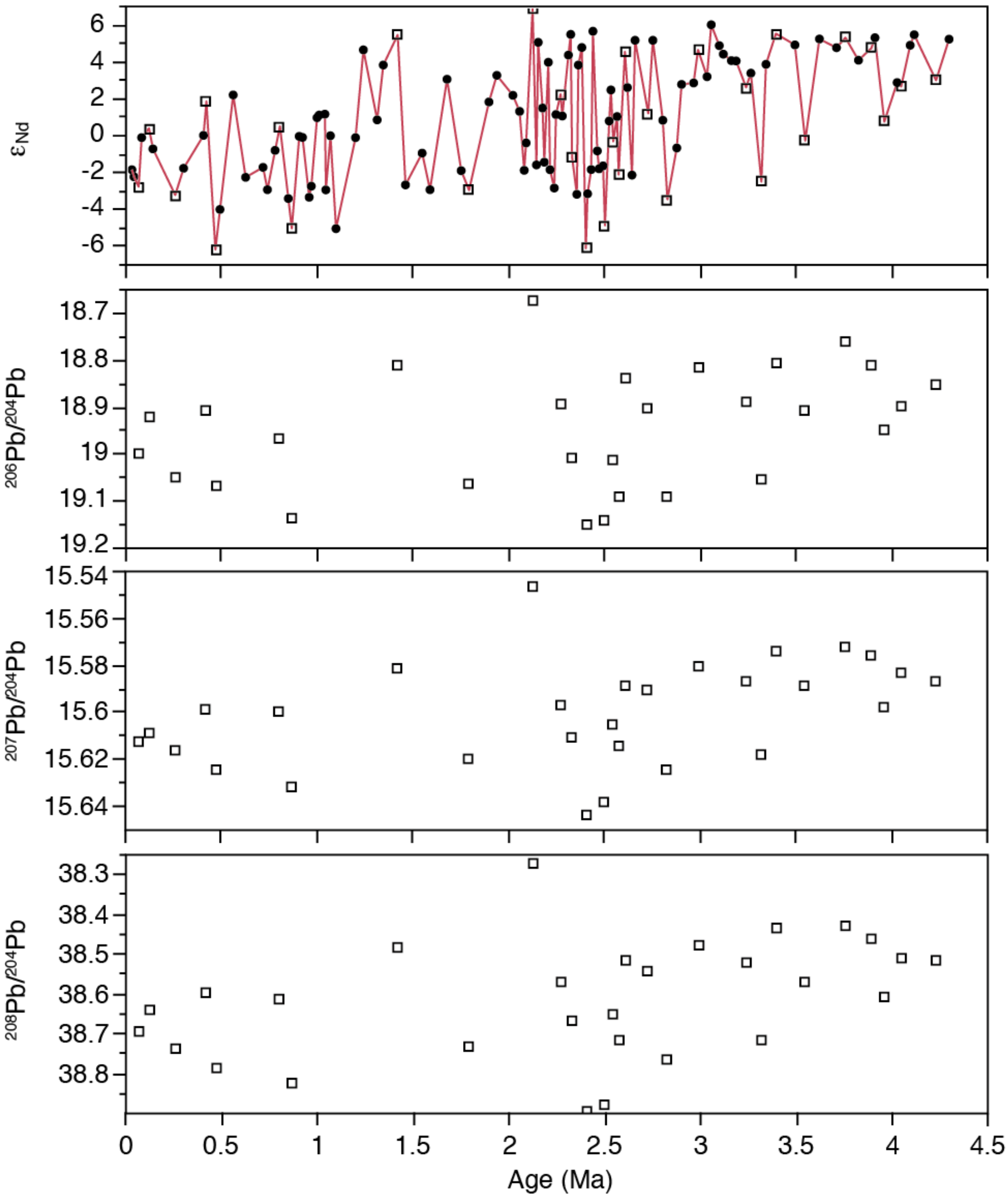

Supplementary Figure 3. Records of ε_{Nd} and Pb isotopic compositions of detrital sediments at Site U1341B in the southern Bering Sea.

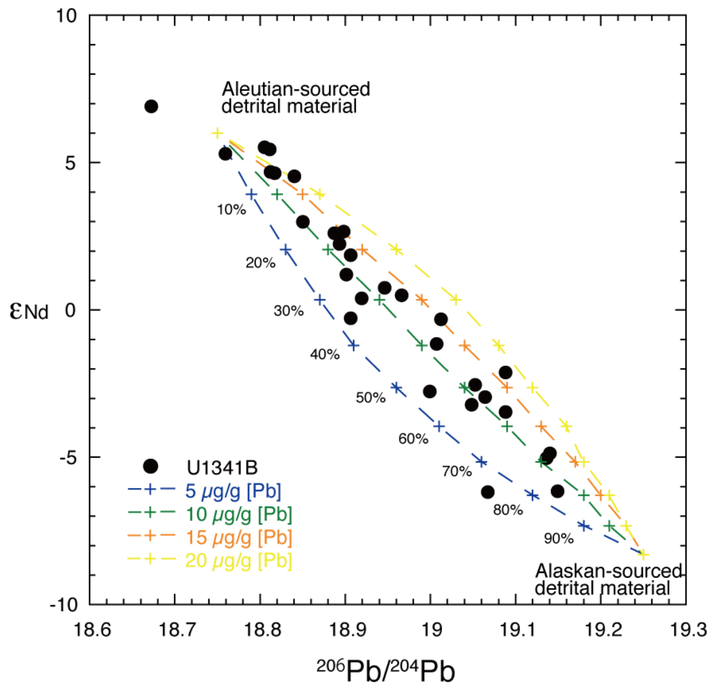

Supplementary Figure 4. Detrital Nd and Pb isotope cross plot. Isotope data from U1341B detrital sediments plotted against four binary mixing models that have different Pb concentrations (5, 10, 15, and 20 µg/g) in the Alaskan-sourced material. The coordinates of points (cross) representing mixtures that have different values of *f* are calculated by the isotope mass-balance equation (Eq. 1).

Supplementary Tables

Supplementary Table 1. Nd and Pb isotope data of detrital sediments from Site U1341B.

Sample ID (Hole, section, depth)	Mid Depth (m)	Age (Ma)	$^{143}Nd/^{144}Nd$	ε_{Nd}	2σ error	$^{208}Pb/^{204}Pb$	2σ error	$^{207}Pb/^{204}Pb$	2σ error	$^{206}Pb/^{204}Pb$	2σ error
U1341B, 1H-3-W, 142-144	4.37	0.031	0.512544	-1.84	0.27						
U1341B, 1H-5-W, 142-144	7.37	0.042	0.512524	-2.22	0.27						
U1341B, 2H-3-W, 142-144	12.95	0.066	0.512496	-2.76	0.27	38.696	0.003	15.612	0.001	18.999	0.001
U1341B, 2H-5-W, 142-144	15.95	0.082	0.512633	-0.10	0.40						
Duplicate1			0.512640	0.04	0.35						
Duplicate2			0.512626	-0.23	0.31						
U1341B, 3H-3-W, 142-144	23.08	0.120	0.512658	0.40	0.27	38.640	0.006	15.609	0.003	18.919	0.003
U1341B, 3H-5-W, 142-144	26.08	0.141	0.512601	-0.71	0.41						
U1341B, 4H-3-W, 142-144	33.38	0.257	0.512473	-3.21	0.27	38.737	0.005	15.616	0.002	19.048	0.002
U1341B, 4H-5-W, 142-144	36.38	0.301	0.512548	-1.76	0.38						
U1341B, 5H-3-W, 141-143	44.05	0.405	0.512639	0.02	0.43						
U1341B, 5H-5-W, 142-144	47.06	0.420	0.512734	1.87	0.27	38.594	0.004	15.599	0.001	18.906	0.002
U1341B, 6H-3-W, 142-144	53.84	0.467	0.512322	-6.17	0.43	38.787	0.004	15.624	0.002	19.067	0.002
U1341B, 6H-5-W, 142-144	56.84	0.491	0.512434	-3.99	0.27						
U1341B, 7H-3-W, 142-144	63.42	0.560	0.512751	2.21	0.27						
U1341B, 7H-5-W, 142-144	66.42	0.625	0.512523	-2.25	0.27						
U1341B, 8H-3-W, 142-144	74.38	0.716	0.512551	-1.71	0.27						
U1341B, 8H-5-W, 142-144	77.38	0.739	0.512489	-2.92	0.27						
U1341B, 9H-3-W, 142-144	84.89	0.779	0.512598	-0.79	0.27						
U1341B, 9H-5, 142-144	87.89	0.804	0.512664	0.51	0.27	38.612	0.005	15.600	0.002	18.966	0.002
U1341B, 10H-3-W, 142-144	94.85	0.848	0.512464	-3.40	0.27						
U1341B, 10H-5-W, 142-144	97.85	0.866	0.512381	-5.02	0.27	38.826	0.004	15.632	0.002	19.136	0.002
U1341B, 11H-3-W, 142-144	104.61	0.905	0.512637	-0.03	0.51						
U1341B, 11H-5-W, 142-144	107.61	0.922	0.512634	-0.09	0.27						
U1341B, 12H-3-W, 142-144	114.52	0.957	0.512467	-3.33	0.27						
U1341B, 12H-5-W, 142-144	117.52	0.968	0.512498	-2.73	0.27						
U1341B, 13H-3-W, 142-144	126.02	0.998	0.512688	0.98	0.27						
U1341B, 13H-5-W, 142-144	129.02	1.009	0.512696	1.12	0.27						
U1341B, 14H-4-W, 146-148	137.41	1.039	0.512698	1.17	0.47						
U1341B, 14H-5-W, 142-144	138.87	1.044	0.512488	-2.93	0.27						
U1341B, 15H-3-W, 142-144	145.40	1.068	0.512638	0.00	0.42						

U1341B, 15H-5-W, 142-144	148.40	1.097	0.512380	-5.03	0.43						
U1341B, 16H-3-W, 142-144	155.87	1.199	0.512633	-0.10	0.47						
U1341B, 16H-5-W, 142-144	158.87	1.240	0.512877	4.65	0.75						
U1341B, 17H-3-W, 142-144	164.20	1.312	0.512682	0.86	0.51						
U1341B, 17H-5-W, 76-78	166.54	1.344	0.512835	3.83	0.27						
U1341B, 18H-3-W, 142-144	172.06	1.419	0.512918	5.46	0.46	38.484	0.004	15.581	0.001	18.811	0.001
U1341B, 18H-5-W, 142-144	175.06	1.460	0.512502	-2.66	0.27						
U1341B, 19H-3-W, 143-145	181.48	1.547	0.512590	-0.94	0.27						
U1341B, 19H-5-W, 143-145	184.48	1.588	0.512489	-2.92	0.27						
U1341B, 20H-3-W, 63-65	191.02	1.677	0.512795	3.06	0.47						
U1341B, 21H-3-W, 142-144	196.40	1.751	0.512541	-1.89	0.27						
U1341B, 21H-5-W, 143-145	199.41	1.792	0.512487	-2.95	0.27	38.732	0.003	15.620	0.001	19.064	0.001
U1341B, 22H-3-W, 142-144	207.10	1.896	0.512732	1.83	0.27						
U1341B, 22H-5-W, 143-145	210.11	1.937	0.512806	3.27	0.54						
U1341B, 23H-3-W, 142-144	216.22	2.021	0.512750	2.19	0.39						
U1341B, 23H-5-W, 142-144	219.22	2.056	0.512706	1.32	0.39						
U1341B, 24H-3-W, 142-144	226.89	2.080	0.512542	-1.87	0.27						
U1341B, 24H-5-W, 142-144	229.89	2.090	0.512618	-0.39	0.39						
U1341B, 25H-5-W, 142-144	241.07	2.124	0.512993	6.92	0.27	38.270	0.004	15.546	0.002	18.672	0.002
U1341B, 26H-3-W, 142-144	247.35	2.144	0.512557	-1.58	0.41						
U1341B, 26H-5-W, 143-145	250.35	2.153	0.512898	5.06	0.27						
U1341B, 27H-3-W, 142-144	257.57	2.176	0.512715	1.50	0.46						
U1341B, 27H-5-W, 142-144	260.57	2.185	0.512565	-1.43	0.27						
U1341B, 28H-3-W, 142-144	267.15	2.206	0.512842	3.98	0.54						
U1341B, 28H-5-W, 142-144	270.15	2.215	0.512544	-1.84	0.47						
U1341B, 29H-3-W, 142-144	277.21	2.237	0.512493	-2.83	0.44						
U1341B, 29H-5-W, 142-144	280.21	2.246	0.512697	1.15	0.44						
U1341B, 30H-3-W, 142-144	287.70	2.270	0.512754	2.25	0.27	38.571	0.003	15.597	0.001	18.893	0.001
U1341B, 30H-5-W, 142-144	290.70	2.279	0.512693	1.07	0.27						
U1341B, 31H-3-W, 142-144	301.07	2.311	0.512862	4.37	0.54						
U1341B, 32H-3-W, 57-59	304.83	2.323	0.512920	5.49	0.27						
U1341B, 32H-5-W, 82-84	307.22	2.330	0.512580	-1.14	0.27	38.667	0.004	15.611	0.001	19.007	0.002
U1341B, 33H-3-W, 142-144	315.06	2.355	0.512476	-3.17	0.27						
U1341B, 33H-5-W, 94-96	317.58	2.363	0.512835	3.83	0.27						
U1341B, 34H-3-W, 143-145	323.47	2.381	0.512883	4.78	0.27						

U1341B, 35H-3-W, 142-144	330.13	2.402	0.512326	-6.10	0.27	38.892	0.004	15.644	0.001	19.149	0.002
Duplicate1			0.512330	-6.01	0.22						
Duplicate2			0.512321	-6.18	0.23						
U1341B, 35H-5-W, 144-146	333.15	2.411	0.512477	-3.14	0.27						
U1341B, 36H-3-W, 144-146	339.15	2.430	0.512544	-1.83	0.27						
U1341B, 36H-5-W, 144-146	342.15	2.439	0.512928	5.66	0.27						
U1341B, 37H-3-W, 144-146	349.48	2.462	0.512596	-0.82	0.27						
U1341B, 37H-5-W, 144-146	352.48	2.471	0.512546	-1.79	0.39						
U1341B, 38H-3-W, 136-138	359.20	2.492	0.512555	-1.63	0.27						
U1341B, 38H-5-W, 144-146	362.34	2.502	0.512389	-4.86	0.39	38.881	0.004	15.638	0.002	19.140	0.002
U1341B, 39H-3-W, 142-144	369.35	2.524	0.512679	0.80	0.27						
U1341B, 39H-5-W, 142-144	372.35	2.533	0.512765	2.48	0.27						
U1341B, 40H-1-W, 142-144	376.18	2.545	0.512622	-0.31	0.51	38.651	0.004	15.605	0.002	19.012	0.002
U1341B, 41H-3-W, 142-144	382.68	2.565	0.512692	1.04	0.68						
U1341B, 41H-5-W, 84-86	385.10	2.573	0.512530	-2.11	0.27	38.717	0.007	15.614	0.003	19.088	0.004
U1341B, 42H-3-W, 142-144	392.18	2.606	0.512871	4.55	0.27	38.513	0.004	15.589	0.002	18.840	0.002
U1341B, 42H-5-W, 83-85	394.59	2.619	0.512772	2.61	0.47						
U1341B, 43H-3-W, 122-124	398.92	2.643	0.512529	-2.13	0.27						
U1341B, 43H-5-W, 132-134	401.24	2.659	0.512903	5.17	0.38						
U1341B, 44H-3-W, 142-144	409.68	2.728	0.512700	1.21	0.40	38.542	0.003	15.590	0.001	18.901	0.001
U1341B, 44H-5-W, 142-144	412.68	2.752	0.512903	5.17	0.46						
U1341B, 45H-3-W, 142-144	419.18	2.804	0.512681	0.84	0.40						
U1341B, 45H-5-W, 132-134	422.08	2.828	0.512461	-3.45	0.27	38.766	0.007	15.624	0.003	19.088	0.004
U1341B, 46H-3-W, 142-144	428.18	2.877	0.512604	-0.66	0.40						
U1341B, 46H-5-W, 142-144	431.18	2.902	0.512781	2.78	0.27						
U1341B, 48H-3-W, 142-144	438.98	2.965	0.512785	2.86	0.27						
U1341B, 48H-5-W, 132-134	441.88	2.988	0.512877	4.65	0.27	38.476	0.005	15.580	0.002	18.817	0.002
U1341B, 50H-3-W, 142-144	447.68	3.035	0.512802	3.20	0.27						
U1341B, 50H-5-W, 111-113	450.37	3.057	0.512946	6.01	0.27						
U1341B, 51H-3-W, 142-144	455.58	3.099	0.512888	4.88	0.46						
U1341B, 51H-5-W, 102-104	458.18	3.120	0.512865	4.42	0.27						
U1341B, 52H-3-W, 142-144	463.38	3.162	0.512847	4.07	0.68						
U1341B, 52H-5-W, 142-144	466.38	3.187	0.512846	4.05	0.27						
U1341B, 53H-3-W, 142-144	472.88	3.239	0.512773	2.62	0.27	38.521	0.003	15.587	0.001	18.887	0.002
U1341B, 53H-5-W, 142-144	475.88	3.264	0.512812	3.39	0.27						

U1341B, 57X-3-W, 141-143	482.77	3.319	0.512511	-2.49	0.27	38.714	0.005	15.618	0.002	19.052	0.002
Duplicate1			0.512515	-2.40	0.19						
Duplicate2			0.512506	-2.57	0.21						
U1341B, 57X-5-W, 131-133	485.67	3.343	0.512837	3.87	0.46						
U1341B, 58X-3-W, 141-143	492.07	3.395	0.512922	5.53	0.27	38.434	0.004	15.574	0.001	18.805	0.002
U1341B, 59X-5-W, 142-144	504.38	3.494	0.512890	4.92	0.45						
U1341B, 60X-3-W, 142-144	511.08	3.548	0.512624	-0.27	0.27	38.572	0.004	15.589	0.002	18.906	0.002
U1341B, 61X-3-W, 142-144	520.58	3.622	0.512907	5.24	0.27						
U1341B, 62X-5, 142-144	533.28	3.711	0.512883	4.77	0.64						
U1341B, 63X-3-W, 142-144	539.98	3.758	0.512910	5.31	0.27	38.430	0.004	15.572	0.001	18.759	0.001
U1341B, 64X-3-W, 142-144	549.58	3.826	0.512848	4.09	0.27						
U1341B, 65X-3-W, 142-144	559.18	3.893	0.512881	4.74	0.27	38.462	0.005	15.576	0.002	18.812	0.002
Duplicate1			0.512880	4.72	0.18						
Duplicate2			0.512882	4.76	0.23						
U1341B, 65X-5-W, 112-114	561.88	3.912	0.512910	5.31	0.27						
U1341B, 66X-3-W, 142-144	568.78	3.961	0.512677	0.76	0.27	38.609	0.006	15.598	0.002	18.946	0.003
U1341B, 67X-3-W, 143-145	578.38	4.028	0.512786	2.88	0.27						
U1341B, 67X-5-W, 142-144	581.38	4.049	0.512775	2.67	0.27	38.512	0.007	15.583	0.003	18.898	0.004
U1341B, 68X-3-W, 142-144	588.08	4.096	0.512889	4.90	0.27						
U1341B, 68X-5-W, 142-144	591.08	4.117	0.512919	5.47	0.42						
U1341B, 70X-3-W, 142-144	607.28	4.231	0.512792	3.00	0.27	38.518	0.004	15.587	0.002	18.850	0.002
U1341B, 71X-3-W, 142-144	616.88	4.299	0.512906	5.23	0.27						

Core depth scale is CCSF-A (m). All reported $^{143}Nd/^{144}Nd$ ratios were corrected for mass fractionation using $^{146}Nd/^{144}Nd$=0.7219. International standard JNdi-1was analyzed between every 3 and 5 unknown samples, and the average of these standard runs was compared to the recommended value of $^{143}Nd/^{144}Nd$ (0.512115)[4] to determine a correction factor for each of the samples analyzed on that day. The $^{143}Nd/^{144}Nd$ ratios are expressed as ε_{Nd} (parts per 10000 variation of $^{143}Nd/^{144}Nd$ ratio relative to the chondritic uniform reservoir[5]). Long-term external reproducibility (2σ) of JNdi-1 analyses on the Nu is ±0.000014 (0.27 ε_{Nd} units). In the case that the internal error was larger than 0.27 ε_{Nd} units, we report the combined internal and external error. Replicate measured samples are indicated by *, which were treated in the different chemical batches and were performed on different days to monitor the reproducibility and accuracy of the isotope data. The precision of replicates was within the 2σ external error. These replicate samples give the mean value.

Supplementary Table 2. Age control points in Site U1341B.

Datums		Depth CCSF-A (m) ± error	Age (Ma) ± error	Reference	Timescale
Benthic foraminifer $\delta^{18}O$		0.45	0.001	This study	
Benthic foraminifer $\delta^{18}O$		2.15	0.018	This study	
Benthic foraminifer $\delta^{18}O$		2.65	0.020	This study	
Benthic foraminifer $\delta^{18}O$		3.16	0.026	This study	
Benthic foraminifer $\delta^{18}O$		6.34	0.038	This study	
Benthic foraminifer $\delta^{18}O$		13.02	0.066	This study	
Benthic foraminifer $\delta^{18}O$		16.75	0.087	This study	
Benthic foraminifer $\delta^{18}O$		21.04	0.108	This study	
Benthic foraminifer $\delta^{18}O$		23.61	0.123	This study	
Benthic foraminifer $\delta^{18}O$		25.30	0.135	This study	
Benthic foraminifer $\delta^{18}O$		27.92	0.156	This study	
Benthic foraminifer $\delta^{18}O$		30.22	0.189	This study	
Benthic foraminifer $\delta^{18}O$		33.04	0.252	This study	
Benthic foraminifer $\delta^{18}O$		35.98	0.295	This study	
Benthic foraminifer $\delta^{18}O$		39.41	0.342	This study	
Benthic foraminifer $\delta^{18}O$		43.10	0.400	This study	
Benthic foraminifer $\delta^{18}O$		49.24	0.432	This study	
Benthic foraminifer $\delta^{18}O$		56.70	0.490	This study	
Benthic foraminifer $\delta^{18}O$		62.49	0.536	This study	
Benthic foraminifer $\delta^{18}O$		65.45	0.611	This study	
Benthic foraminifer $\delta^{18}O$		66.72	0.630	This study	
Benthic foraminifer $\delta^{18}O$		66.96	0.644	This study	
Benthic foraminifer $\delta^{18}O$		68.62	0.662	This study	
Benthic foraminifer $\delta^{18}O$		72.79	0.697	This study	
Benthic foraminifer $\delta^{18}O$		74.56	0.718	This study	
Benthic foraminifer $\delta^{18}O$		76.11	0.732	This study	
Benthic foraminifer $\delta^{18}O$		76.84	0.738	This study	
Benthic foraminifer $\delta^{18}O$		80.41	0.746	This study	
Benthic foraminifer $\delta^{18}O$		86.97	0.795	This study	
Benthic foraminifer $\delta^{18}O$		88.82	0.813	This study	
Diatom	LCO *Actinocyclus oculatus*	112.48 ± 2.06	0.95 ± 0.05	Onodera et al (in press)	CK95
Paleomagne	Jaramillo Bottom	146.57	1.07	Takahashi et al (2011)	ATNTS2004
Diatom	LCO *Neodenticula koizumii*	218.75 ± 1.51	2.06 ± 0.05	Onodera et al (in press)	ATNTS2004
Paleomagne	Gauss Top	387.75	2.58	Takahashi et al (2011)	ATNTS2004
Diatom	LO *Neodenticula kamtschatica*	400.10 ± 1.16	2.65 ± 0.05	Onodera et al (in press)	CK95
Paleomagne	Gilbert Top	516.95	3.60	Takahashi et al (2011)	ATNTS2004
Diatom	FO *Neodenticula koizumii*	555.90 ± 3.87	3.87 ± 0.02	Onodera et al (in press)	ATNTS2004

Referenced age datums (not included in our age model for core U1341B)

Diatom	LO *Proboscia curvirostris*	34.90 ± 1.51	0.30	Onodera et al (in press)	CK95
Diatom	LO *Thalassiosira jouseae*	37.67 ± 1.28	0.35 ± 0.05	Onodera et al (in press)	CK95
Silicoflagellate	LO *Dictyocha subarctios*	93.49 ± 1.75	0.74	Onodera et al (in press)	ATNTS2004
Paleomagne	Brunhes Bottom	87.76	0.78	Takahashi et al (2011)	ATNTS2004
Paleomagne	Jaramillo Top	109.98	0.99	Takahashi et al (2011)	ATNTS2004
Diatom	FO *Proboscia curvirostris*	170.27 ± 1.82	1.58	Onodera et al (in press)	Barron (2003)
Ebridian	LO *Ebriopsis antiqua antiqua*	345.84 ± 3.66	2.48	Onodera et al (in press)	ATNTS2004
Silicoflagellate	LO *Distephanus jimlingii*	370.81 ± 0.30	2.52	Onodera et al (in press)	ATNTS2004

Diatom and paleomagnetic datums are from Onodera et al[6] and Takahashi et al[7]. Geologic timescales CK95, ATNTS2004, and Barron (2003) are from ref. 8 ref. 9 and ref. 10, respectively.

Supplementary Table 3. The Nd and Pb isotopic compositions and their concentrations in Alaskan and Aleutian arc rocks used for the isotope mass balance calculation.

	εNd	Nd (μg/g)	^{206}Pb/^{204}Pb	Pb (μg/g)
Alaskan-sourced detrital material	-8.3	18.3	19.25	10
Aleutian-sourced detrital material	6	12	18.75	7

The end-member values of Nd and Pb (^{206}Pb/^{204}Pb) isotopic compositions and their concentrations were estimated from relevant literature[11,12] and the GEOROC database[13].

Supplementary Note 1

Alaskan sediment supply from the Yukon River

The Yukon River drains an area of land measuring approximately 853,300 km^2, and flows for a length of approximately 3340 km from northwestern Canada through Alaska, USA–making it the longest river in the Bering Sea[14] (Fig. 1). The southern portion of its drainage basin includes the glacier-covered high mountains of the Alaska Range, Wrangell Mountains, and the St. Elias Mountains. Seasonal melting of mountain glaciers and snow yields abundant suspended sediments in the Tanana and White Rivers during the spring and summer seasons[14]. As a result, sediments transported to the Bering Sea are mainly (90 wt.% or more) composed of silt- and clay-sized materials[15]. The annual export of suspended sediments from the Yukon River is estimated to be 55 Mt/yr[16], which represents the highest amount of sediment load transported into the Bering Sea; the next two largest sediment loads are from the Kuskokwim River (8 Mt/yr) in Alaska and the Anadyr River in Russia (2 Mt/yr)[17]. Both of these sediment loads are relatively minor detrital sources in the Bering Sea. As explained in the text, the Nd and Pb isotopic compositions of detrital sediments from U1341B create linear arrays that connect Aleutian arc material and Yukon-Tanana terrane Alaskan material, suggesting the significant influence of the Yukon River-delivered Alaskan detrital inputs to the southern Bering Sea (Fig. 3).

In this study, we also analyzed the 20-ka records of detrital Nd isotopes from the well-dated sediment core (Bow-8A) from Bowers Ridge (Fig. 1). The ε_{Nd} values represent glacial–interglacial variations, including radiogenic ε_{Nd} values during the Holocene and less radiogenic values during the last deglaciation and the glacial period (Supplementary Fig. 2). It is considered that this glacial–interglacial variability in detrital ε_{Nd} values may be related to the growth and decay cycles of the Bering/Alaskan cryosphere. Less radiogenic ε_{Nd} values during the last glacial and deglacial periods can be explained by enhanced Yukon River discharge associated with deglacial meltwater from retreating ice sheets and mountain glaciers in Alaska and ice-rafted debris during glacial period, respectively. A comparison of detrital ε_{Nd} values with benthic foraminiferal δ^{18}O records from site U1341B also shows that such glacial–interglacial variability in the detrital ε_{Nd} values can be seen for a period of time spanning at least the past 1.0 Ma (Supplementary Fig. 2).

Supplementary References

1. Okada, M., Takagi, M., Narita, H. & Takahashi, K. Chronostratigraphy of sediment cores from the Bering Sea and the subarctic Pacific based on paleomagnetic and oxygen isotopic analyses. *Deep Sea Research Part II* **52,** 2092–2109 (2005).
2. Horikawa, K., Asahara, Y., Yamamoto, K. & Okazaki, Y. Intermediate water formation in the Bering Sea during glacial periods: Evidence from neodymium isotope ratios. *Geology* **38,** 435–438 (2010).
3. Lisiecki, L. E. & Raymo, M. E. A Pliocene-Pleistocene stack of 57 globally distributed benthic $\delta^{18}O$ records. *Paleoceanography* **20**, doi:10.1029/2004PA001071 (2005).
4. Tanaka, T. *et al.* JNdi-1: a neodymium isotopic reference in consistency with LaJolla neodymium. *Chem. Geol* **168**, 279–281 (2000).
5. Jacobsen, S. B. & Wasserburg, G. J. Sm-Nd isotopic evolution of chondrites. *Earth Planet. Sci. Lett.* **50,** 139–155 (1980).
6. Onodera, J., Takahashi, K. & Nagatomo, R. Diatoms, silicoflagellates, and ebridians at Site U1341 on the western slope of Bowers Ridge, IODP Expedition 323. *Deep Sea Res. Part II Top. Stud. Oceanogr.*, doi:10.1016/j.dsr2.2013.03.025 (in press).
7. Takahashi, K., Ravelo, A. C., Alvarez Zarikian, C. A. & the Expedition 323 Scientists. Proc. IODP 323, Tokyo (Integrated Ocean Drilling Program Management International, Inc.). doi:10.2204/iodp.proc.323.2011 (2011).
8. Cande, S. C. & Kent, D. V. Revised calibration of the geomagnetic polarity timescale for the Late Cretaceous and Cenozoic. *J. Geophys. Res.* **100,** 6093–6095 (1995).
9. Lourens, L., Hilgen, F., Shackleton, N. J., Laskar, J. & Wilson, D. in *A Geological Time Scale* (eds F.M. Gradstein, J.C. Ogg, & A.G. Smith), Cambridge University Press, p. 409–440 (2004).
10. Barron, J. A. Planktonic marine diatom record of the past 18 M.Y.: Appearances and extinctions in the Pacific and Southern Oceans. *Diatom Research* **18,** 203–224 (2003).
11. Asahara, Y. *et al.* Provenance of terrigenous detritus of the surface sediments in the Bering and Chukchi Seas as derived from Sr and Nd isotopes: Implications for recent climate change in the Arctic regions. *Deep Sea Res. Part II Top. Stud. Oceanogr.* **61-64,** 155–171 (2012).
12. Aleinikoff, J. N., Lang Farmer, G., Rye, R. O. & Nokleberg, W. J. Isotopic evidence for the sources of Cretaceous and Tertiary granitic rocks, east-central Alaska: implications for the tectonic evolution of the Yukon–Tanana Terrane. *Can. J. Earth Sci* **37,** 945–956 (2000).
13. GEOROC database. at http://georoc.mpch-mainz.gwdg.de/georoc/.
14. Dornblaser, M. M. & Striegl, R. G. Suspended sediment and carbonate transport in the Yukon River Basin, Alaska: Fluxes and potential future responses to climate change. *Water Resour. Res.* **45**, doi:10.1029/2008wr007546 (2009).
15. Chikita, K. A., Kemnitz, R. & Kumai, R. Characteristics of sediment discharge in the subarctic Yukon River, Alaska. *CATENA* **48,** 235–253 (2002).

16 Eberl, D. D. Quantitative mineralogy of the Yukon River system: Changes with reach and season, and determining sediment provenance. *Am. Mineral.* **89,** 1784–1794 (2004).

17 VanLaningham, S., Pisias, N. G., Duncan, R. A. & Clift, P. D. Glacial–interglacial sediment transport to the Meiji Drift, northwest Pacific Ocean: Evidence for timing of Beringian outwashing. *Earth Planet. Sci. Lett.* **277,** 64–72 (2009).
